# Th2‐Polarised CD4^+^ T Cells Enhance *Staphylococcus aureus* Growth in a 3D Skin Model

**DOI:** 10.1111/cea.70019

**Published:** 2025-02-19

**Authors:** Ina Suhrkamp, Melina Fonfara, Magdalena Magdalena, Jan N. Hartmann, Elke Rodriguez, Jürgen Harder, Hila Emmert, Stephan Weidinger

**Affiliations:** ^1^ Department of Dermatology and Allergy University Hospital Schleswig‐Holstein, Campus Kiel Kiel Germany; ^2^ Research Unit of Molecular Epidemiology Helmholtz Zentrum München Neuherberg Germany

**Summary**

- Treatment with IL‐4/IL‐13 remarkably alters the epidermal organisation in a way that is uniquely consistent with atopic dermatitis in 3D skin models, highlighting the potential of this therapy in addressing skin inflammation associated with this condition.
- The inclusion of immune cells is essential for effectively mimicking the heightened growth of *S. aureus* .

**Abbreviations**
3D: three dimensional
AD: atopic dermatitis
AMP: antimicrobial peptides
CD: cluster of differentiation
Dpl: dupilumab
IL: interleukin

*S. aureus*

:
*Staphylococcus aureus*

Th: T helper

To the editor,

Atopic dermatitis (AD) is characterised by a skin barrier defect, type 2 immune‐mediated inflammation, and microbial dysbiosis [1]. Three‐dimensional human skin equivalents (3DSE) effectively mimic human skin by providing a stratified barrier grown on a dermis‐like matrix. These models enable mechanistic AD studies; however, few have incorporated bacteria or immune cells, both of which are essential to fully recapitulate AD features. In this study, we refined AD‐like 3DSEs by integrating *Staphylococcus aureus* ( *S. aureus* ) and immune cells to more accurately simulate AD pathology.

3DSEs were generated following established protocols [2]. An AD‐like environment was created by stimulation with IL‐4 and IL‐13 or Th2‐polarised CD4^+^ T cells, and *S. aureus* was applied topically. Biopsies from the 3DSEs were taken for histological assessment, bacterial DNA and human RNA isolation, and the basolateral media were collected for multiplex ELISA. Additional information about study methods and findings is available in the following repository: https://zenodo.org/records/14773303.

IL‐4 and IL‐13 were used to stimulate simple 3D skin models of AD. These models showed a reduced expression of filaggrin, involucrin and loricrin compared to unstimulated models (Figure 1A). Cytokine stimulation further induced spongiosis, indicating a profound effect of IL‐4/IL‐13 on skin barrier integrity (Figure 1B). The inflammatory milieu in the basolateral media of IL‐4/IL‐13 models was marked by an overall increase in levels of pro‐inflammatory cytokines (Figure 1D).

**FIGURE 1:**
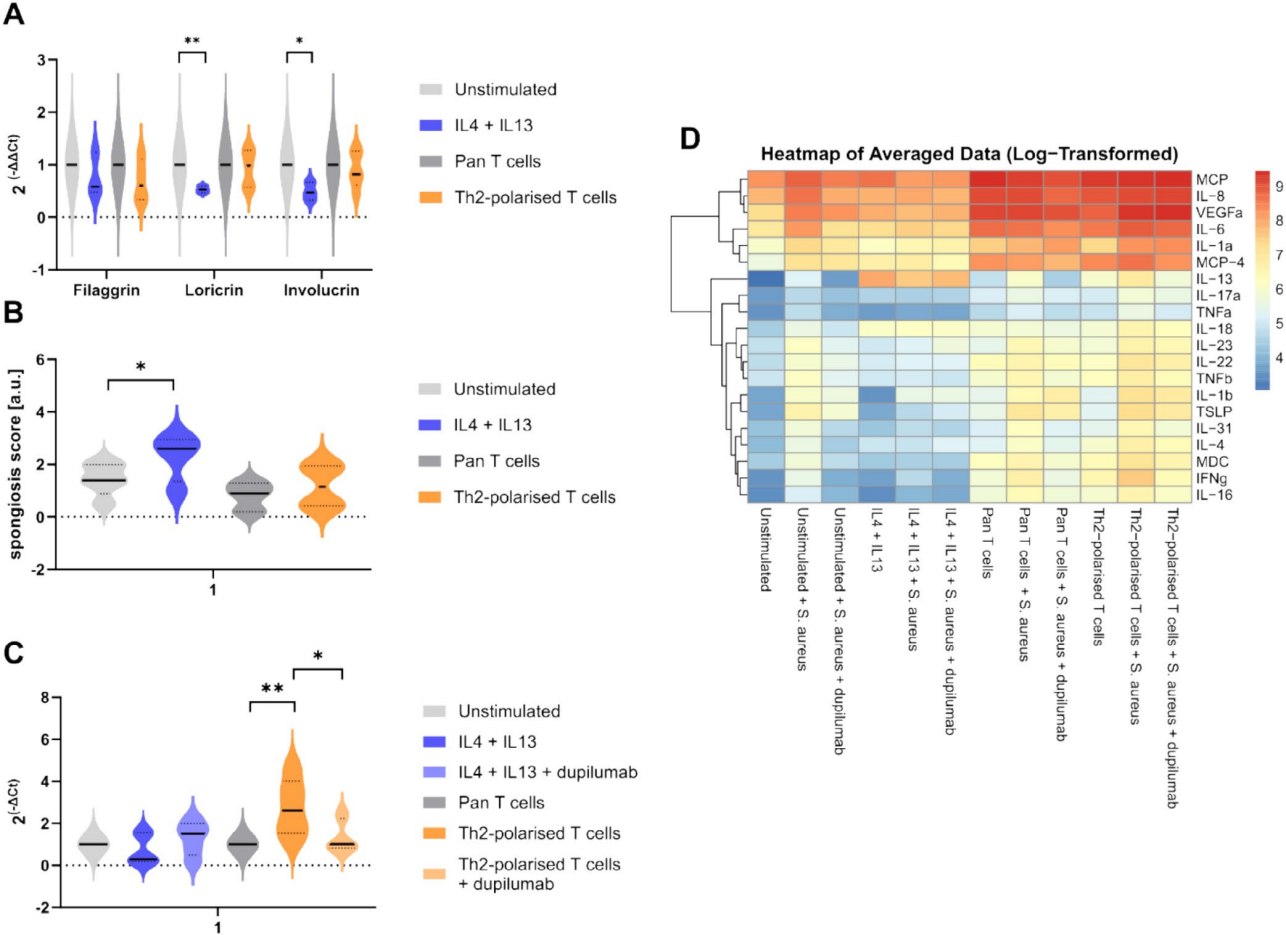
Changes in epidermal organisation, inflammatory milieu and *Staphylococcus aureus* ( *S. aureus* ) abundance in 3D skin equivalents after stimulation with IL‐4 and IL‐13 or T cells. (A) RNA was isolated from a 4 mm punch biopsy and real‐time PCR was performed in order to assess gene expression of selected skin barrier proteins. ∆∆ Ct values were calculated using RPL38 as housekeeping gene and normalised to control models (unstimulated or pan T cell treated). One sample t‐test was performed to find statistically significant differences between stimulated and respective control groups. (B) Spongiosis scoring performed on H&E‐stained histological slides. Subjective scoring was performed by 5 different researchers. Scoring range was 0–3. Paired *t*‐test was performed to find statistically significant differences between stimulated and respective control groups. (C) Bacterial DNA was isolated from a 4 mm punch biopsy and real‐time PCR was performed for quantification. Ct values of *S. aureus* grown on control 3D skin equivalents (either unstimulated or pan T cell stimulated) served as reference. Kruskal–Wallis and post hoc Dunn's test with Benjamini‐Hochberg correction were performed to find statistically significant differences between the groups. (D) Basolateral medium was collected and levels of pro‐inflammatory mediators were assessed using multiplex ELISA. Kruskal–Wallis and post hoc Dunn's test with Benjamini–Hochberg correction were performed to find statistically significant differences between the groups. At least three independent experiments have been performed. *p ≤ 0.05; **p ≤ 0.01.

The colonisation of skin with *S. aureus* presents an important factor in AD pathology, as 90% of atopic dermatitis patients, but only 5% of healthy individuals, show *S. aureus* skin colonisation [3]. Whether the changes in skin morphology are a cause or consequence of increased growth of *S. aureus* on skin is widely discussed. In clinical settings, dupilumab, a systemic treatment targeting IL4Rα, not only ameliorates AD symptoms but can also reduce pro‐inflammatory cytokine levels and has been shown to be associated with reduced *S. aureus* skin colonisation [4]. Our addition of *S. aureus* on our simple 3DSEs aimed to mimic the in vivo‐observed increase in *S. aureus* load. However, cytokine treatment did not increase *S. aureus* load; neither did dupilumab reduce bacterial growth (Figure 1C). Although inducing defects in skin barrier integrity, IL‐4/IL‐13‐stimulated 3DSEs failed to mimic the effects observed in vivo regarding systemic therapy and microbial dysbiosis. Thus, in the current 3DSEs, the in vivo effects cannot be fully mimicked.

In recent years, 3D skin models became more advanced by the integration of immune cells [5]. However, these models did not consider the analysis of skin microbial imbalances. We integrated in vitro Th2‐polarised CD4^+^ T cells into our 3D skin equivalents to more accurately replicate the in vivo conditions of AD. For control comparisons, non‐polarised CD4^+^ T cells from healthy donors (pan T cells) were used. The indirect incorporation of Th2‐polarised CD4^+^ T cells into the basolateral media led to decreased expression of filaggrin and involucrin in the skin models, with minor effects on loricrin expression and the development of spongiosis (Figure 1A,B). Although skin barrier defects were less severe in these T cell models, an increased *S. aureus* load compared to respective control skin models was observed as opposed to cytokine‐treated models (Figure 1C). Treatment with dupilumab effectively reduced the increased *S. aureus* growth in models including Th2‐polarised cells. Our results indicate that immunocompetent 3D skin equivalents seem to be essential to recapitulate the increased *S. aureus* growth observed in AD lesional skin, suggesting that the Th2 milieu is crucial for the induction of *S. aureus* growth in 3D skin models.

Th2 or pan T cell integration both generated a cytokine‐rich environment compared to simple models, which was even more enriched when skins were exposed to *S. aureus* . We observed elevated levels of IL‐4, IL‐13, IL‐1α, IL‐1β, IFN‐γ, IL‐16, IL‐22, IL‐17A and MDC (Figure 1D). Whether these cytokines were expressed by T cells or skin resident cells like keratinocytes or fibroblasts is not known. The observed induction of IL‐1α and IL‐1β by *S. aureus* in our models is consistent with previous studies [6], suggesting this pathway might play an important role in *S. aureus* ‐mediated skin barrier impairment. Brauweiler et al. showed that *S. aureus* ‐derived lipoteichoic acid reduces filaggrin and involucrin expression and that this reduction is mediated via an IL‐1‐mediated pathway [7]. Most of the identified pro‐inflammatory proteins appear to be a consequence of the increased *S. aureus* load, as they were further increased after the addition of *S. aureus* . For instance, IL‐17A and IL‐22 induce AMP production, which should protect against *S. aureus* colonisation [8]. Innate immunity markers such as IL‐1β are secreted by keratinocytes to promote defence by inducing AMP production [9]. IL‐22, detectable in AD lesional skin, induces AMP production but also causes downregulation of epidermal differentiation complex genes, resulting in enhanced *S. aureus* colonisation [8]. Thus, colonisation by *S. aureus* may induce the skins defence mechanisms, but unknown factors exist by which *S. aureus* can continue to propagate.

Our results represent a major step forward in the development of complex skin models, including microbial and immunological parameters, with the potential for further enhancement by incorporation of different members of the skin microbiome and other immune players. For future studies, it is crucial to carefully consider the research question to select the appropriate 3D skin model. IL‐13 + IL‐4 treated 3D skin equivalents seem to be better suited for assessing changes in epidermal organisation and skin barrier impairment, while 3D skin models incorporating Th2 cells are more appropriate for evaluating changes in *S. aureus* load.

## Author Contributions

Conceptualization: I.S., M.F., H.E. Data curation: I.S., M.F., M.M. Formal analysis: I.S., M.F., H.E. Funding acquisition: S.W. Investigation: I.S., M.F., M.M. Methodology: I.S., M.F., H.E., J.H., M.M. Project administration: S.W., H.E. Resources: S.W., H.E., J.H. Supervision: H.E., J.H., E.R. Validation: I.S., M.F., H.E. Visualisation: J.N.H., M.F. Writing – original draft: I.S., M.F. Writing – review and editing: H.E., S.W., J.H., J.N.H., E.R., M.M. All authors have read and agreed to the final version.

## Conflicts of Interest

S. Weidinger has received institutional research grants from Sanofi Deutschland GmbH, LEO Pharma, and Pfizer and performed consultancies and/or lectures for AbbVie, Almirall, Boehringer, Eli Lilly, Galderma, Kymab, Leo Pharma, Regeneron, Sanofi‐Genzyme, and Novartis. H. Emmert and J. Harder have received institutional research grants from LEO Pharma. The rest of the authors declare that they have no conflicts of interest.

## Data Availability

The data that support the findings of this study are available from the corresponding author upon reasonable request.
